# CRISPR/Cas9 knockout of female-biased genes *AeAct-4* or *myo-fem* in *Ae*. *aegypti* results in a flightless phenotype in female, but not male mosquitoes

**DOI:** 10.1371/journal.pntd.0008971

**Published:** 2020-12-18

**Authors:** Sarah O’Leary, Zach N. Adelman

**Affiliations:** 1 Department of Entomology, Texas A&M University, College Station, Texas, United States of America; 2 Interdisciplinary Program in Genetics, Texas A&M University, College Station, Texas, United States of America; University of Cincinnati, UNITED STATES

## Abstract

*Aedes aegypti* is a vector of dengue, chikungunya, and Zika viruses. Current vector control strategies such as community engagement, source reduction, and insecticides have not been sufficient to prevent viral outbreaks. Thus, interest in novel strategies involving genetic engineering is growing. Female mosquitoes rely on flight to mate with males and obtain a bloodmeal from a host. We hypothesized that knockout of genes specifically expressed in female mosquitoes associated with the indirect flight muscles would result in a flightless female mosquito. Using CRISPR-Cas9 we generated loss-of-function mutations in several genes hypothesized to control flight in mosquitoes, including actin (*AeAct-4*) and myosin (*myo-fem*) genes expressed specifically in the female flight muscle. Genetic knockout of these genes resulted in 100% flightless females, with homozygous males able to fly, mate, and produce offspring, albeit at a reduced rate when compared to wild type males. Interestingly, we found that while *AeAct-4* was haplosufficient, with most heterozygous individuals capable of flight, this was not the case for *myo-fem*, where about half of individuals carrying only one intact copy could not fly. These findings lay the groundwork for developing novel mechanisms of controlling *Ae*. *aegypti* populations, and our results suggest that this mechanism could be applicable to other vector species of mosquito.

## Author summary

*Ae*. *aegypti* is the most important vector of arboviruses throughout the world, and new strategies are urgently needed to add to our existing arsenal of control methods to prevent or halt disease transmission. Importantly, only female *Ae*. *aegypti* mosquitoes transmit arboviruses due to their need for vertebrate blood to support egg production. Here, we identify several genes that are critical only for female mosquito flight; genetic ablation of each gene resulted in flightless females, but flying males. Importantly, the female flightless phenotype was completely penetrant (100%), and the use of sex-biased flight muscle proteins appeared to be conserved throughout mosquito evolution. These data could be used to support the development of novel genetic control approaches to reduce the number of biting females across a wide range of disease vector species.

## Introduction

The yellow fever mosquito *Aedes aegypti* is a vector for many viruses of medical significance, such as dengue, Zika, chikungunya, and yellow fever, and they can be found in tropical, subtropical, and temperate regions of the world [1]. Only female *Ae*. *aegypti* bite to obtain a blood meal, which is required for egg production. After hatching from the embryo, *Ae*. *aegypti* like all other mosquito species will progress through the aquatic larval and pupal stages of their life, before emerging as an adult from the pupal casing to fly away [2,3].

Due to a lack of safe, effective vaccines for most viruses transmitted by *Ae*. *aegypti*, save yellow fever virus [4], control efforts largely focus on reducing vector abundance, and include source reduction, and chemical methods like insecticides or larvicides [3,5]. The short-term effect and high financial cost, along with the need for trained staff, presents challenges to the implementation, scaling, and maintenance of these control methods [3,6]. With chemical methods, additional concerns relating to the emergence of resistance and effects on off-target species are increasing [6]. Because of these limitations, the need for novel vector control strategies is growing.

Genetic control strategies are receiving an increased amount of attention as viable vector control approaches, and include sterile insect technique (SIT) [7–12], release of an insect carrying a dominant lethal (RIDL) [7,10,13], and potentially gene drive [14–23]. Gene drive involves the spread of a genetic element beyond Mendelian rates of inheritance [14,23–25]. Synthetic gene drive mechanisms can take advantage of the CRISPR/Cas9 system, which allows targeting of the genome at a precise location to catalyze a double-stranded break with repair outcomes (non-homologous end joining or homology directed repair) determining whether the result is targeted disruption or copying of a cargo sequence. Population suppression approaches to vector control with genetic modifications seek to, in some way, prevent the female mosquito from being able to bloodfeed or mate, thus producing fewer or no offspring and leading to a population decline or collapse [15,20–22]. Population replacement can couple a cargo, such as refractoriness to a pathogen, with a gene drive to potentially replace the native vector population with a new population less capable of transmitting the pathogen [16–19]. Much work is being put in to understanding the formation of alleles resistant to CRISPR/Cas9 cleavage [20,21,26–29] and to increasing gene drive efficiencies overall [22,27,28,30–32]. Meanwhile, the CRISPR/Cas9 system has now become an efficient and inexpensive method for genome editing [33], and it has been utilized effectively in *Ae*. *aegypti* for both genome editing [34–38] and gene drive [38].

A function that is critical for both reproduction and survival in female mosquitoes is flight, as flight is required for mating, obtaining a blood meal, and escaping from aquatic breeding sites after eclosion. *AeAct-4* was identified previously as a female- and pupal-specific gene, with expression in the indirect flight muscles [39]. Other work in *Ae*. *aegypti* has identified a male-specific actin gene [40] and male-specific myosin gene [41] related to flight, while in *Drosophila melanogaster*, Flightin has been identified as a flight-associated protein that is hypothesized to function in the indirect flight muscles of both sexes by interacting with myosin filaments or by modulating actin-myosin interactions [42]. Knockout of the male-specific myosin gene has shown that it is needed for male flight [43], and offers up the possibility that male and female flight in *Aedes* mosquitoes is controlled separately by these sex-specific genes.

To determine the importance of selected actin and myosin genes to *Ae*. *aegypti* female flight, we used CRISPR/Cas9 to generate heritable loss-of-function alleles in *AeAct-4* and a female-biased myosin gene which we termed *myo-fem*, along with a third gene, *Aeflightin*, that is expressed in both males and females. Phenotypic analysis of individuals homozygous for each introduced mutation in *AeAct-4* or *myo-fem* confirmed that flight defects were both complete and restricted to females. Males homozygous for either mutation were capable of mating and producing viable progeny. While *AeAct-4* knockout males could fly and mate, their ability to compete for females was reduced compared to wild-type males; this was not the case for loss of *myo-fem*. Disruption of *Aeflightin* was associated with loss of flight in both sexes. Phylogenetic analysis of *AeAct-4* suggests that other mosquito species genomes likely contain female- and male-biased actin genes as well. These data support the pursuit of novel genetic strategies geared specifically for disrupting female flight in *Ae*. *aegypti* and other vector species of mosquito.

## Materials and methods

### Insect rearing

The Liverpool strain of *Ae*. *aegypti* was used for embryonic microinjections and outcrossing of mutant individuals. All mosquitoes were reared at 28°C and 75–80% humidity, with a 14/10 h light/dark light cycle. Ground up fish food (Tetra, Blacksburg, VA) was supplied throughout the aquatic developmental stages, and a cotton ball soaked with 10% sucrose solution was supplied during the adult stage. Flightless mosquitoes were supplied with raisins as the source of sucrose. Defibrinated sheep blood (Colorado Serum Company, Denver, CO) was offered for blood feeding via a parafilm membrane feeder. Videos were taken with a Canon Rebel T3i digital camera.

### Computational analysis

Publicly available mapped RNA-seq data from Akbari et al. (2013) was retrieved from VectorBase [44]. Raw counts for each gene of interest and all paralogs with ≥80% amino acid similarity were obtained using featureCounts using only uniquely mapped reads [45]. Raw counts data were linear normalized based on transcript length and library size to obtain fragments per kilobase per million reads (FPKM) data. FPKM data were transformed to avoid negative values: [log10(1+FPKM)]. Transformed data were used to generate a heat map with Morpheus (Morpheus, https://software.broadinstitute.org/morpheus). A maximum score of 3.8 was set when generating the heat map based on the highest expression value across all samples/timepoints.

For phylogenetic and molecular evolutionary analyses, VectorBase [44] and FlyBase [46] were used to perform a BLASTP search for orthologs of *AeAct-4* and all paralogs with ≥80% amino acid similarity (maximum e-value 1e^-3^, word size 3). All sequences obtained were aligned with MUSCLE [47] and compared using the Neighbor-Joining method in MEGA version X [48]. Evolutionary distances were computed using the Poisson correction method and are in the units of the number of amino acid substitutions per site. All ambiguous positions were removed for each sequence pair (pairwise deletion option), with a total of 376 positions in the final dataset.

All bar graphs were generated, and Chi square analyses performed, using GraphPad Prism (version 8 for Windows, GraphPad Software, La Jolla, CA, www.graphpad.com).

### Guide RNA design and synthesis

Guide RNAs were designed by hand with the DNASTAR SeqBuilder Pro software (Madison, WI), using the appropriate gene sequence acquired through VectorBase [44]. Primers used to make each sgRNA were ordered through IDT. Guide RNA synthesis was performed as previously described [34]. Briefly, Q5 High-Fidelity DNA Polymerase (New England BioLabs Inc., Ipswich, MA) was used for the PCR reaction, followed by the NucleoSpin Gel and PCR Clean-Up kit protocol (Machery-Nagel, Bethlehem, PA), the MEGAscript T7 Transcription kit protocol, and the MEGAclear Transcription Clean-Up kit protocol (Thermo Fisher Scientific, Waltham, MA). All sgRNAs were quantified, aliquoted, and stored at -80°C. A list of oligos used to make each sgRNA are listed in S2 Table.

### Embryo microinjections

The generation and identification of knockout strains followed essentially from our previously published protocols [49]. Briefly, borosilicate glass capillaries (World Precision Instruments Inc., Sarasota, FL) were pulled and beveled using the Sutter P-2000 Micropipette Puller and Sutter BV-10 Micropipette Beveller (Sutter Instrument Co., Novato, CA). Embryo microinjections were performed using a Leica DM 1000 LED Micromanipulator (Leica Biosystems, Buffalo Grove, IL) and FemtoJet 4i Microinjector (Eppendorf, Hauppauge, NY). Purified Cas9 protein (400 ng/μl) (PNA Bio, Thousand Oaks, CA) and sgRNAs (100 ng/μl) were combined into injection mixes, incubated at 37°C for 30 minutes, and centrifuged at max speed (18,213 g), 4°C, for a minimum of 45 minutes. Injections were performed into the posterior end of embryos that were less than three hours old. “*AeAct-4* Exp. 1” injection mixes included *kmo* sgRNAs, while “*AeAct-4* B Exp. 2” injection mixes included only site B sgRNAs. Injected embryos were either harvested at 24 hours (for embryo assays) or hatched after five days. Hatched G_0_ survivors were outcrossed to either the wild type Liverpool strain (for *AeAct-4* and *myo-fem*) or the *kmo* knockout strain (for *Aeflightin*). Mutant males (n = 10–25) were selected at each subsequent generation for continued outcrossing through G_3_ (for *Aeflightin*) or G_5_ (for *AeAct-4* and *myo-fem*) before intercrosses were performed.

### DNA extraction, PCR, HRMA, and Sanger sequencing

Genomic DNA was extracted from non-injected or sgRNA-injected Liverpool embryos following the Nucleospin Tissue kit protocol (Machery-Nagel, Bethlehem, PA). Embryo assays were performed with the LightScanner Master Mix kit (Idaho Technology Inc., Salt Lake City, UT), and mutant detection was performed on adult legs with the Phire Animal Tissue Direct PCR kit (Thermo Fisher Scientific, Waltham, MA) with added LCGreen Plus+ Melting dye (Idaho Technology Inc., Salt Lake City, UT). All samples were amplified with the C1000 Touch Thermal Cycler (Bio-rad, Hercules, CA) before being analyzed with the LightScanner Call-IT 2.0 software on the LightScanner instrument (Idaho Technology Inc., Salt Lake City, UT). Sanger sequencing was performed at the Laboratory for Genomic Technologies (Institute for Plant Genomics and Biotechnology, Texas A&M University, College Station, TX) and chromatograms were analyzed using Chromas software (Technelysium, Australia). A list of primers used are located in S1 Table.

### Flight determination

To assess flight, pupae were placed in plastic ketchup containers (with the stick of a cotton swab or Q-tip to aid in escaping from the water due to defects in flight) in 5-quart plastic buckets (Home Depot) designed with a mesh covering over the top and a sock for internal access on the side. The plastic lining of the bucket inhibited the mosquitoes from climbing up the sides towards the mesh covering. Flying mosquitoes that could fly up to the mesh covering were removed from the plastic bucket, and 24 hours was allowed to pass after the last pupae emerged before classifying the remaining mosquitoes as flightless. Dead adults whose flight phenotype could not be confirmed were not analyzed for a genotype.

### Mating competition assays

To assess mating competitiveness between wild type and homozygous mutant (*AeAct-4* and *myo-fem*) males, 40 WT/HOM males (based on HRMA analysis) were placed into a 170 oz container with 40 Liverpool females for mating. After at least 24 hours post-mating, the females were offered a blood meal for a minimum of 30 minutes, and engorged females were selected and separated from males. The EAgaL plate fecundity and fertility assay protocol was followed [50]. Briefly, a single blood fed female was placed in each well of a 24-well plate that had been prepared by filling each well with agarose. Females were placed at least 72 hours after blood feeding and were given up to 48 hours to deposit embryos. After 48 hours, females were removed, water was added to the wells, and the embryos were monitored over the next 5 days for hatching. If present, 10 or more larval progeny were collected from each individual female that produced embryos. Amplicons derived from the *AeAct-4* or *myo-fem* target sequence from both the male parents and the pooled larval progeny were sequenced to determine the respective starting and resulting genotypes, and thus identify the genotype of the male that mated with the female. At least three biological replicates per mutant strain were performed.

## Results

Since female mosquitoes rely on flight to obtain a blood meal and mate, disrupting flight specifically in females could prevent both reproduction and the transmission of arboviruses. AeAct-4 has been characterized as a female- and pupal-specific flight protein that is expressed in the indirect flight muscles [39]. Indeed, re-analysis of RNA-seq data from the developmental transcriptome of *Ae*. *aegypti* [51] confirmed that expression of *AeAct-4* was highly biased towards female pupae (S1 Fig). Of the eight paralogous actin genes, six showed expression throughout development and in pupae of both sexes, one (AAEL005964) was pupae-, but not sex-specific, and one (AAEL009451) was expressed specifically in male pupae. AAEL005656 is a paralog to the gene *myo-sex* (AAEL021838), a male-specific myosin gene located in the M locus on chromosome one [41] and required for male flight [43]. As AAEL005656 was found to be expressed primarily in female pupae (S1 Fig), we reasoned that it might be similarly critical for female flight, and refer to this gene as *myo-fem*. Finally, AAEL004249 is a 1:1 ortholog of *Drosophila melanogaster*, *flightin*, which has been shown to be expressed in the indirect flight muscles [42], with knockout resulting in a loss of flight ability [52]. We refer to this gene as *Aeflightin* and confirm that it is expressed almost exclusively in pupae in both males and females (S1 Fig). We reasoned that despite lacking female specificity, *Aeflightin* may be a good target for disrupting female flight, so long as a male-specific rescue can be performed.

For each of these three target genes, we designed multiple groups of overlapping sgRNAs, described in S2 Table with either “site” or “exon” for each group. Due to high nucleotide sequence similarity between *AeAct-4* and other actin paralogs, we performed a multiple sequence alignment prior to sgRNA design. Eight paralogs with ≥80% nucleotide sequence identity to *AeAct-4* were aligned to attempt to identify sgRNA targets that were unique to *AeAct-4*. Candidate guide RNA sequences were identified in regions where there was more sequence variability between *AeAct-4* and the paralogs (S2 Fig). For *myo-fem*, guide RNAs were designed to target the motor domain to ensure disruption of myosin function. For *Aeflightin*, guide RNAs were designed to each exon as we did not identify any paralogous genes in the *Ae*. *aegypti* genome.

**Fig 1:**
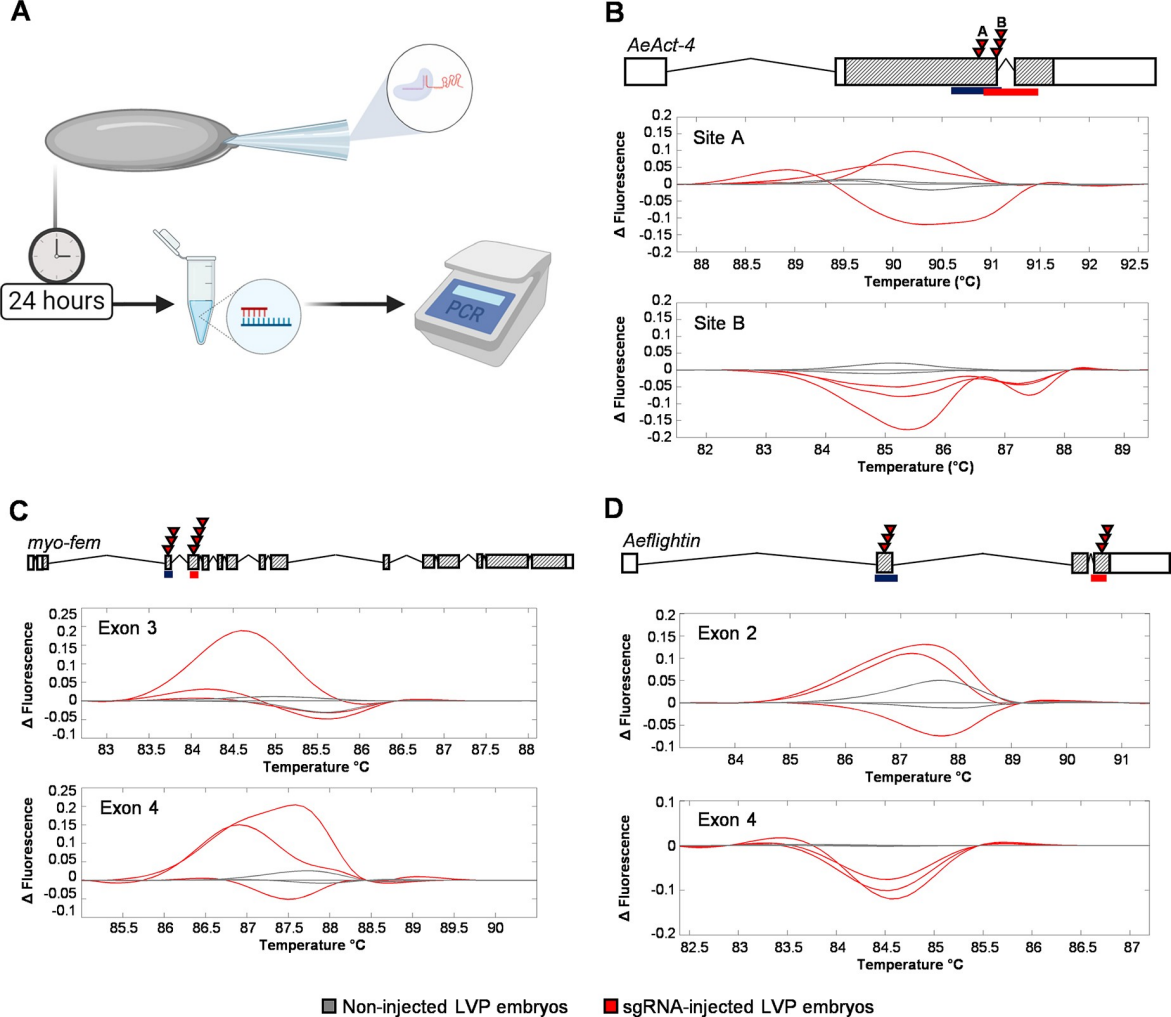
Development of CRISPR reagents targeting *Ae*. *aegypti* genes involved in flight. (**A**) Workflow for performing embryo assays. Gene models and HRMA analysis following embryonic microinjection of CRISPR/Cas9 reagents with each group of sgRNAs for *AeAct-4* (**B**), *myo-fem* (**C**), and *Aeflightin* (**D**). For (**B-D**), boxes represent exons, while cross-hatched areas represent the ORF of the corresponding mRNA. For each, red triangles indicate the locations of sgRNAs that were found to successfully cleave the DNA during embryo assay, and the blue and red boxes under the gene models indicate the approximate HRMA amplicon sizes. Melt curves are displayed for clusters of sgRNAs (indicated as “site” or “exon”), with sgRNA-injected (red) and non-injected (gray) samples.

Following in vitro synthesis, sgRNAs were complexed with Cas9 protein and injected in groups of 3–4 into *Ae*. *aegypti* embryos, which were harvested after 24 hours. To identify those gRNA batches capable of inducing strong gene disruption, genomic DNA from injected embryos was used as a template for PCR and HRMA of the target region (Fig 1A). For *AeAct-4* (Fig 1B), *myo-fem* (Fig 1C), and *Aeflightin* (Fig 1D), we identified two clusters of guide RNAs for each gene with detectable editing in embryos.

**Fig 2:**
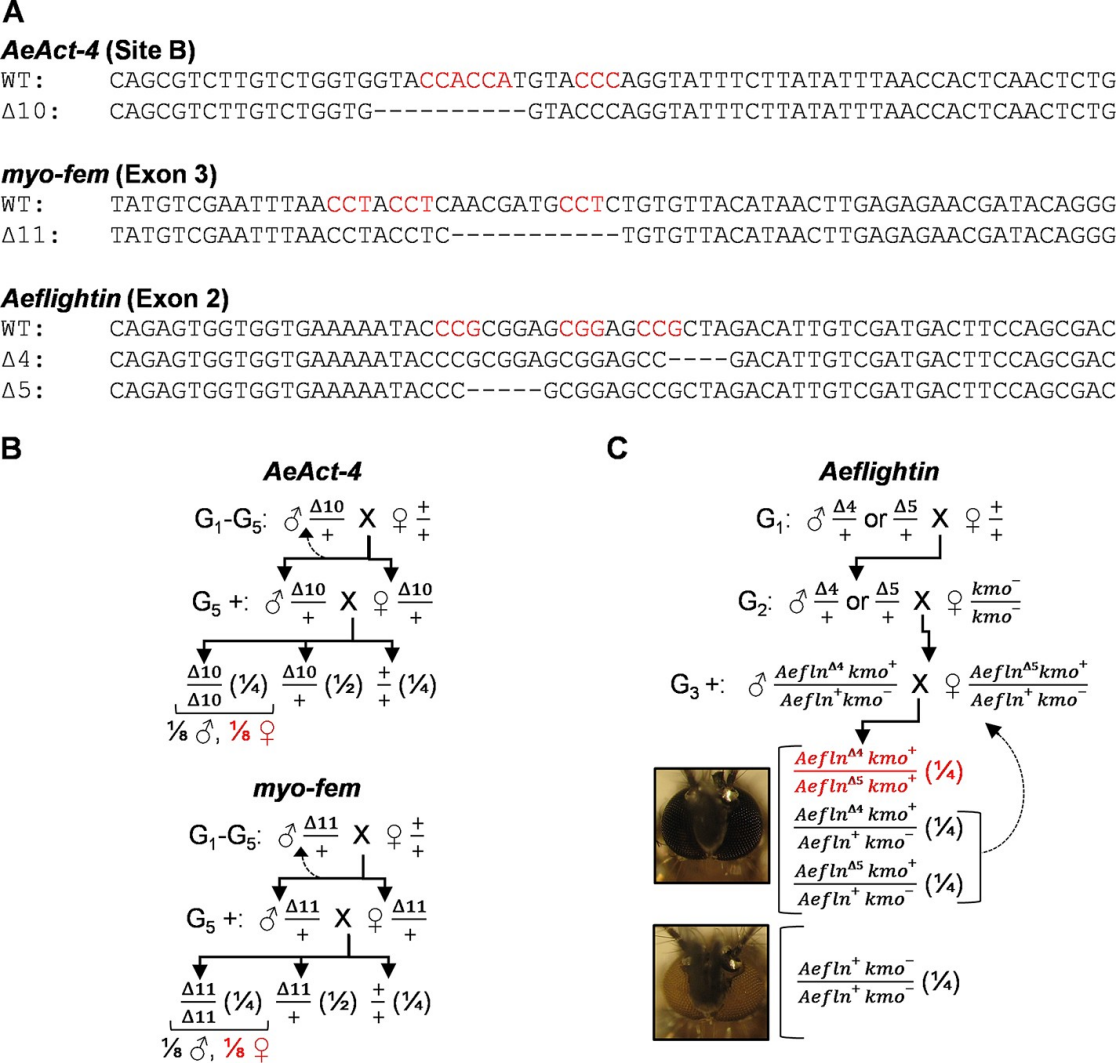
Establishment and maintenance of heritable loss-of-function mutations in *Ae*. *aegypti* flight genes. (**A**) For each gene, the wild type (WT) and mutant sequence is shown. The PAM sites for each sgRNA used in the injection mix for the specified location are highlighted in red. Individuals containing each deletion were outcrossed through the indicated generation, at which point individuals heterozygous for the *AeAct-4*, *myo-fem* (**B**), and *Aeflightin* (**C**) mutations were intercrossed. For each cross, the ratio of each potential genotype expected is noted in parentheses, with the individuals with an expected flightless phenotype highlighted in red. For *Aeflightin*, phenotypic identification of all white-eyed pupae enables their removal before further phenotypic analysis based on flight (see Fig 4) is performed.

**Fig 3:**
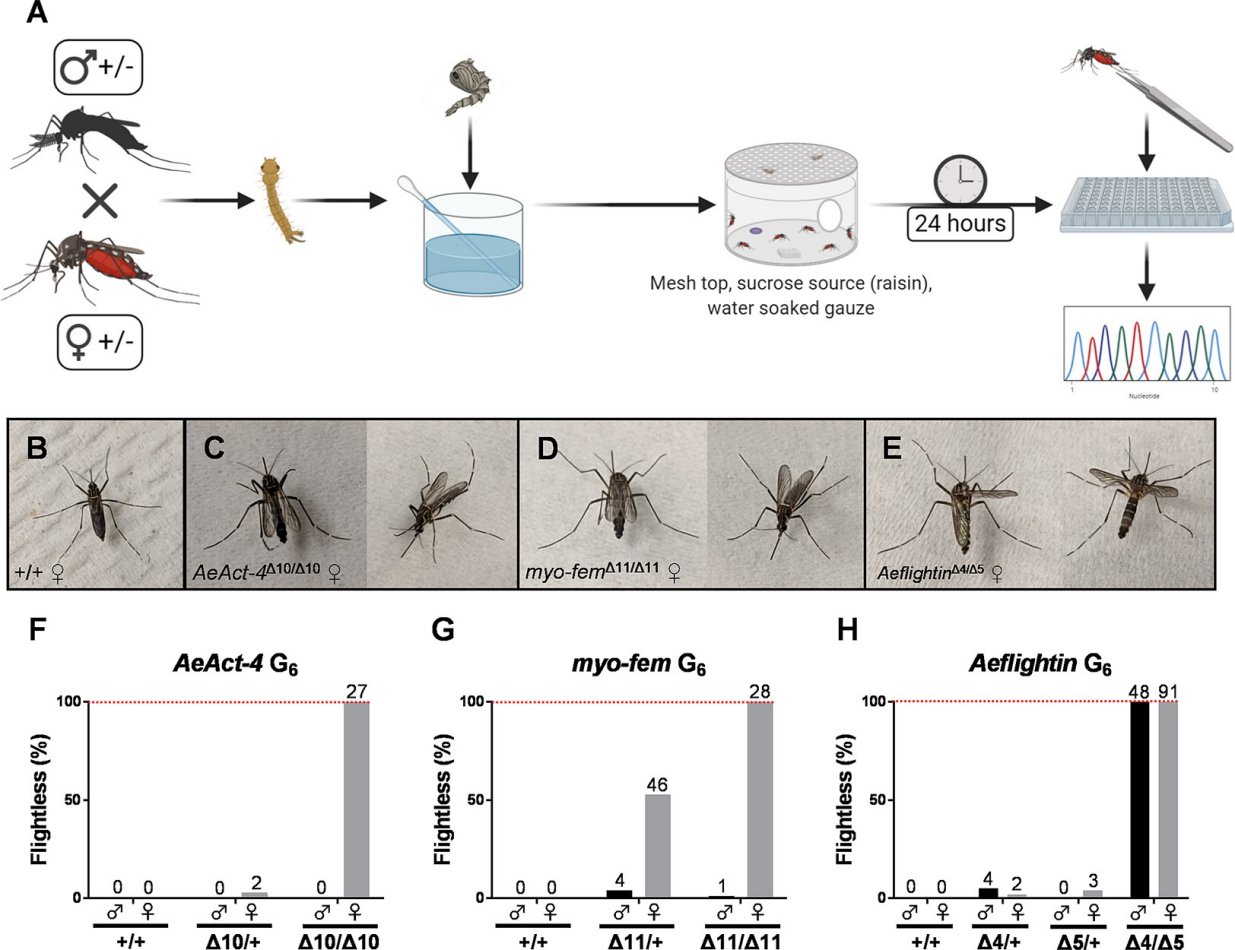
Loss of *AeAct-4*, *myo-fem*, or *Aeflightin* results in flightlessness. (**A**) Blinded workflow used to score flight ability without experimenter knowledge of genotype, with subsequent genotyping assays. Photographs of wild type (**B**), *AeAct-4* ^Δ10/Δ10^ (**C**), *myo-fem* ^Δ11/Δ11^ (**D**), or *Aeflightin*^Δ4/Δ5^ (**E**) females when resting. The percentage of flightless male (black bars) or female (grey bars) mosquitoes for *AeAct-4* (**F**), *myo-fem* (**G**), and *Aeflightin* (**H**) based on each genotype. The number above each bar represents the number of individuals displaying the flightless phenotype and were confirmed for the specified genotype via sequencing. The dotted red line indicates 100% flightless.

For each mixture of guide RNAs that displayed editing activity, we repeated embryo microinjections and this time allowed the embryos to hatch after five days. Survivors were outcrossed to the parental Liverpool strain to obtain G_1_ progeny. G_1_ adults were screened via PCR, HRMA, and Sanger sequencing for out-of-frame mutations (S3 Table). Deletions predicted to result in a frameshift mutation were recovered for *AeAct-4*, *myo-fem*, and *Aeflightin* (Fig 2A). Genotyped males with out-of-frame mutations (n = 10–25) were outcrossed to females from the parental strain for three (for *Aeflightin*) or five (for *AeAct-4* and *myo-fem*) generations, followed by intercrosses of heterozygous individuals (Fig 2B and 2C). Backcrossing of the mutant strains was performed to reduce any CRISPR/Cas9 off-target effects and assist in the recovery from the genetic bottleneck associated with single founder events. *AeAct-4* is located on chromosome three, as are three other actin paralogs (AAEL001928, AAEL005961, and AAEL005964). Therefore, we performed Sanger sequencing of each actin paralog to confirm that there were no off-target effects in these paralogs linked to *AeAct-4*, as these may have been maintained despite backcrossing (S3 Fig). G_6_ progeny from heterozygous intercrosses were characterized as flightless or flying (Fig 3A and S4 Table). Upon stimulation of flight, control mosquitoes could fly, while some from each test cross could not; these were therefore categorized as flightless and hypothesized to be homozygous for each targeted gene disruption. Flightless individuals appeared to have various alternative wing phenotypes when resting that differed from wild type (Fig 3B–3E). Some individuals could move or beat their wings, but with no success in flight. Other individuals could initiate flight takeoff, but not sustain flight. Agitation to provoke flight via shaking or tapping of the plastic buckets (S1–S3 Videos), or gentle spraying of condensed air were used to evaluate flight ability; flightless mosquitoes remained so regardless of the method of stimulation.

As all observations of flight behavior were made without consideration for genotype, we sought to determine whether there was a relationship between the ability of mosquitoes to fly and inheriting one or two copies of each loss-of-function mutation. A genotypic analysis of all flightless individuals and a subset of flying individuals was performed for *AeAct4* and *myo-fem* (S4 Table and Fig 3F and 3G) as well as *Aeflightin* (S5 Table and Fig 3H). All *AeAct-4*^*Δ10/Δ10*^ males and all *myo-fem*^*Δ11/Δ11*^ males except one (S4 Table) could fly (*AeAct-4* = 100%; *myo-fem* = 96%), while all *AeAct-4*^*Δ10/Δ10*^ and *myo-fem*^*Δ11/Δ11*^ females were flightless (100%). Interestingly, while most heterozygous *AeAct-4*^*Δ10/+*^ females could fly (97%, only two were flightless), this was not the case for *myo-fem*^*Δ11/+*^, where about half of females (53%) were categorized as flightless. The flying to flightless ratio did not differ significantly from the expected ratio for *AeAct-4* (p = 0.6503, Chi square analysis), suggesting the flightless phenotype was completely recessive in this case. Due to the presence of a substantial number of flightless heterozygous females for *myo-fem* (46 individuals) as compared to *AeAct-4* (2 individuals), the flying to flightless ratio was significantly different from the expected ratio (p < 0.0001, Chi square analysis), suggesting that defects in *myo-fem* are not entirely recessive. From the intercross between *AeAct-4*^*Δ10/+*^ parents, we observed that male genotype ratios of WT, heterozygous, and homozygous individuals did not differ from the null expectation, suggesting a lack of strong fitness cost to males associated with the mutation (p = 0.6628, Chi square analysis). In contrast, we observed a deficiency of homozygous males and a corresponding excess of heterozygotes for *myo-fem* (p = 0.0008, Chi square analysis). As the *Aeflightin* gene is tightly linked to the *kmo* gene involved in eye pigmentation, *Aeflightin* mutants were outcrossed to a kynurenine 3-monooxygenase (*kmo*) knockout strain [34] to help track the corresponding genotypes. This aided in phenotypic identification of homozygous *kmo* individuals who do not carry the *Aeflightin* mutation, as well as maintenance of a transheterozygous line (Fig 2C). At G_6_, recombination between *kmo* and *Aeflightin* was observed at 1–3%, consistent with expectations (S5 Table). After scoring flight phenotypes, genotypes were determined by HRMA and sequencing. Critically, we found that all transheterozygous *Aeflightin*^*Δ4/Δ5*^ individuals were flightless, confirming that *Aeflightin* is required for flight in both male and female *Ae*. *aegypti* (Fig 3 and S5 Table). The flying to flightless ratio did not differ significantly from the expected ratio for *Aeflightin* (p = 0.5319, Chi square analysis), suggesting that a single copy of *Aeflightin* is sufficient to program flight, and the associated flightless phenotype is recessive.

**Fig 4:**
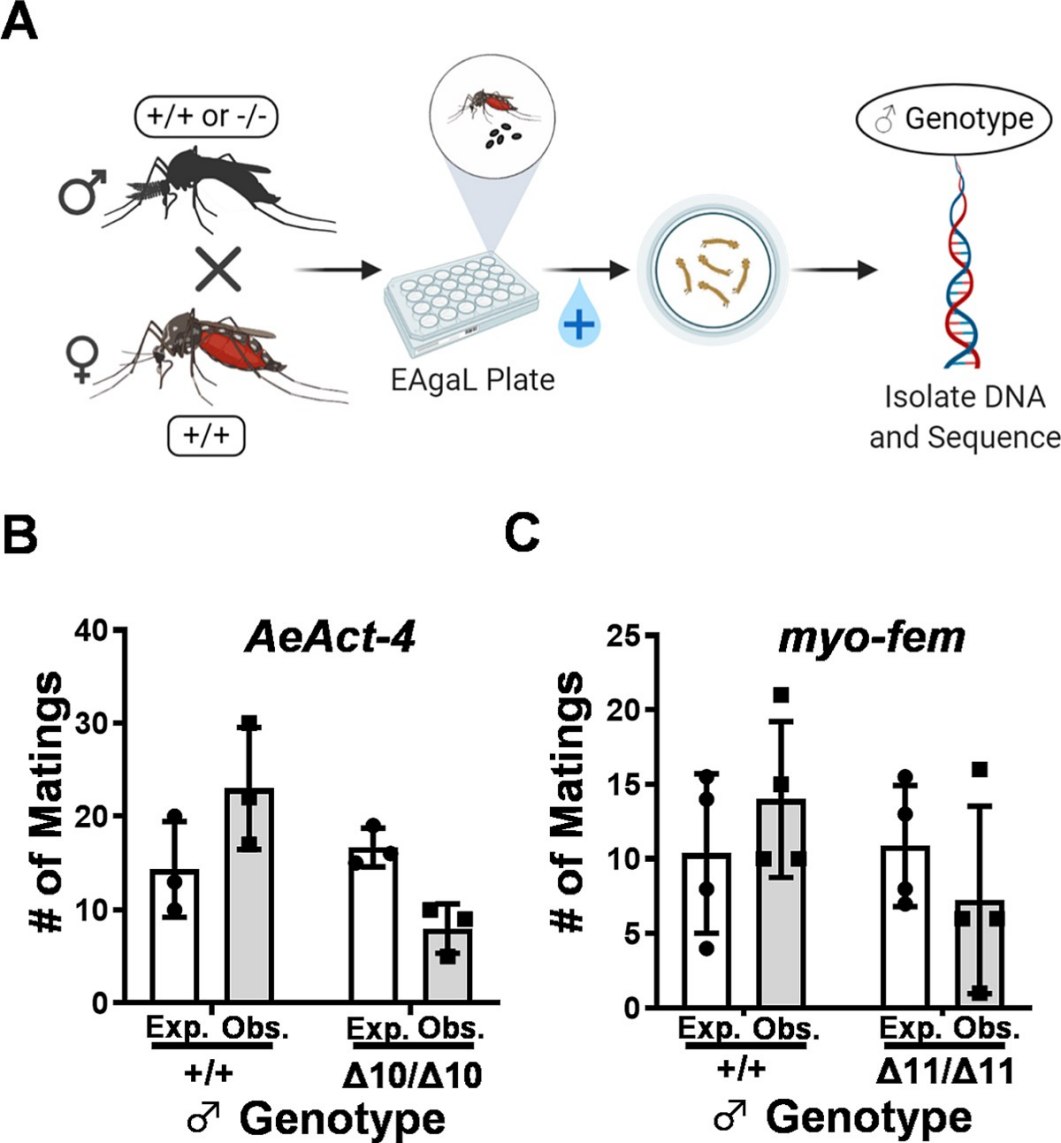
Mating competitiveness of male *Ae*. *aegypti* deficient in *AeAct-4* or *myo-fem*. (**A**) Workflow for performing larval progeny assays. The observed (Obs.) vs. expected (Exp.) number of matings for *AeAct-4* (**B**) and *myo-fem* (**C**) males based on the sequenced genotypes of pooled larval progeny obtained from the male mating competitiveness assays. Wild type is defined as +/+ and homozygous mutants are defined as Δ10/Δ10 (for *AeAct-4*) or Δ11/Δ11 (for *myo-fem*). Each data point represents one replicate, bar height represents the mean of all replicates, and the error bars indicate standard deviation. Chi square was performed for statistical analysis.

As we noted that both *AeAct-4* and *myo-fem* are expressed at low levels in male pupae [51] (S1 Fig), we hypothesized that this may indicate these genes contribute to male flight. While our loss-of-function data suggest these gene products are not required for flight, we reasoned that they may still contribute to male mating success, which occurs in flight. Thus, we sought to compare the mating competitiveness between wild type and homozygous mutant males, an important consideration if these genes are to be potential targets in a genetic control approach such as gene drive. We took advantage of the fact that wild type and homozygous individuals cannot be distinguished by HRMA alone in our assay (requires sequencing) to perform a series of blinded mating competition experiments. After screening by HRMA, 40 males with WT/HOM genotypes were crossed with 40 wild type females. After 1–3 days, females were offered a bloodmeal and progeny were collected from each female individually. At the same time, all HRMA amplicons were sequenced to obtain the starting percentage of each male genotype. In the case of a mating between a WT male and a WT female, all progeny would be WT, while matings between homozygous mutant males and WT females should result in progeny heterozygous for the mutation (either *AeAct-4*^*Δ10*^ or *myo-fem*^*Δ11*^*)*. HRMA and sequencing analysis was used to determine the genotype of the larval progeny, and thus, the genotype of the individual male that mated with that female (Fig 4A). Wild type males significantly outperformed *AeAct-4*^*Δ10/Δ10*^ males in mating with wild type females (Fig 4B; *AeAct-4* p = 0.0018, Chi square analysis), suggesting that *AeAct-4* may contribute to the mating success of male *Ae*. *aegypti*. In contrast, for *myo-fem* (Fig 4C) the difference in mating success was not significantly different from expectations based on starting genotypes (*myo-fem* p = 0.0805, Chi square analysis). We note that we did not monitor the females for remating events, thus it is possible that mutant individuals are less competitive than indicated. We also note that for *myo-fem*, there was more variation in the data than for *AeAct-4*, and this may serve to obscure real differences in competitiveness. Data for each replicate experiment is presented individually (S6 Table), along with the expected and observed larval progeny genotypes.

**Fig 5:**
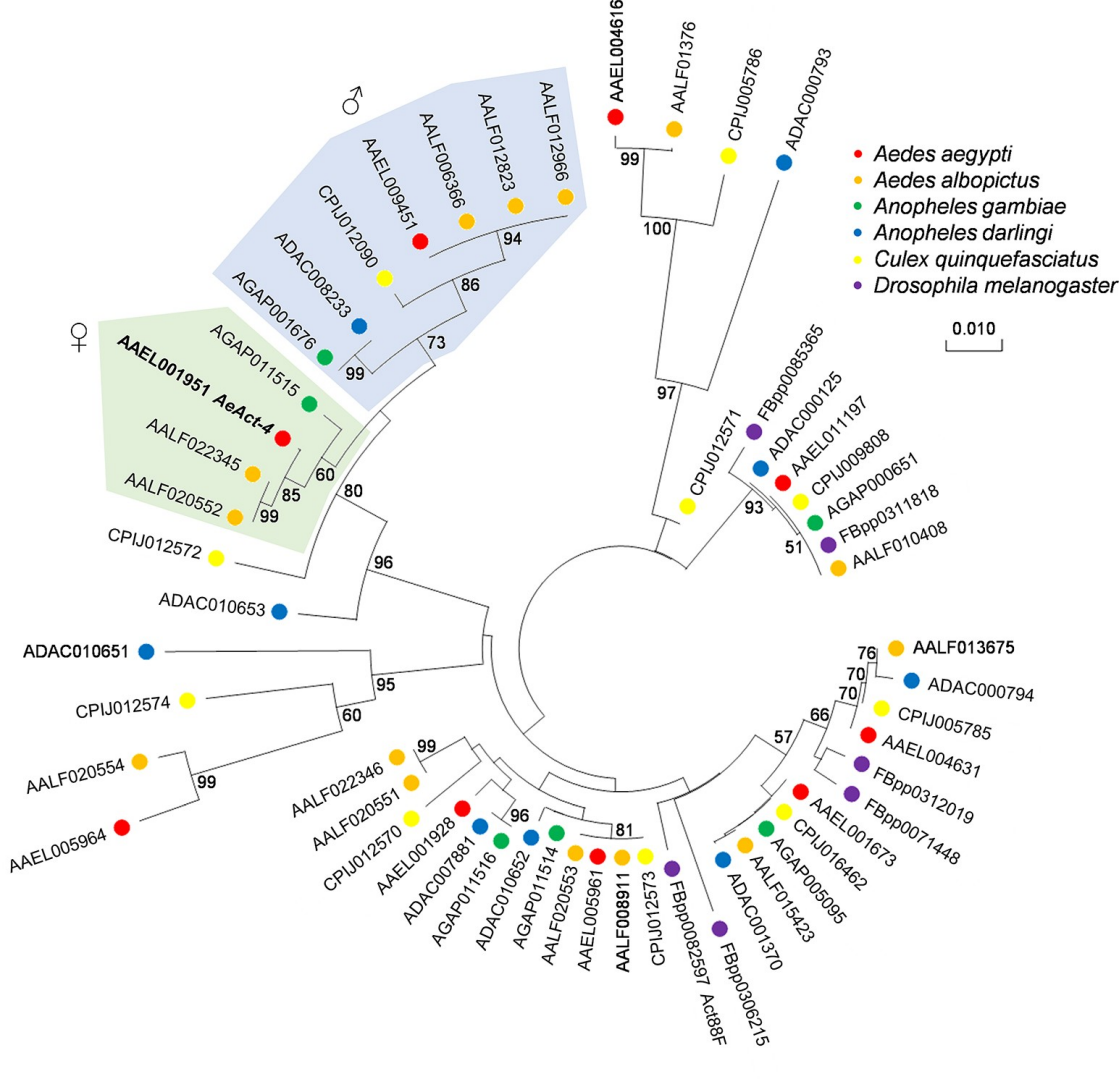
Neighbor-joining tree of *AeAct-4* and related homologs in mosquitoes. A phylogenetic analysis of *AeAct-4* (in bold face) and all paralogs with ≥80% amino acid similarity in mosquitoes and *Drosophila*. The gene identifiers include *Ae*. *aegypti* (AAEL in red), *Ae*. *albopictus* (AALF in orange), *An*. *gambiae* (AGAP in green), *An*. *darlingi* (ADAC in blue), *Cu*. *quinquefasciatus* (CPIJ in yellow), and *D*. *melanogaster* (FBpp in purple). Female-specific genes are represented in the green shaded area, and male-specific genes are in the blue shaded area. All branch points with >50% support based on 1,000 bootstrap replicates are indicated.

After successfully knocking out flight-associated genes in *Ae*. *aegypti*, we were interested in finding out if other mosquito genomes contain sex-specific flight genes. Previous phylogenetic analysis of the male-specific *myo-sex* suggests that both male- and female-specific myosin flight genes evolved in *Aedes* after divergence from *Culex* [41]. To determine if this was also the case for *AeAct-4*, we performed a phylogenetic analysis of actin protein sequences across multiple mosquito species, as well as the fruit fly *Drosophila melanogaster* (Fig 5). Both *AeAct-4* and the male-biased actin gene AAEL009451 clustered separately with orthologs from *Ae*. *albopictus*, *Culex*, and *Anopheles* mosquitoes, suggesting a more ancient origin to these sex-biased genes. Consistent with this reconstruction, the *An*. *gambiae* gene AGAP011515 grouped with *AeAct-4* and was previously found to be preferentially expressed in females [53], while AGAP001676 groups with the *Aedes* male actin gene, and was also found to have significantly increased expression in the male carcass (head, thorax, and abdomen, excluding the reproductive tissue) as compared to the female carcass [54]. Together, these data suggest that genetic approaches targeting flight specifically in females may be broadly applicable across mosquito genera.

## Discussion

Our results indicate successful knockout of three flight-specific genes, two of which are expressed predominantly in females (*AeAct-4* and *myo-fem*). Interestingly, we found that while *AeAct-4* and *Aeflightin* were haplosufficient, two intact copies of *myo-fem* appeared to be required for normal female flight. We note though that our approach allowed 24 hours after the last adult emerged for all mosquitoes to gain the ability to fly. For *myo-fem*, there were a few individuals who subsequently gained the ability to fly up to 48 hours after all flyers had been removed. These individuals seemed to have a delay (≥ 24 hours post-eclosion) in gaining flight ability, suggesting that a single copy of *myo-fem*, while insufficient to program the normal timing of development of the flight muscles, may be sufficient provided the female can survive long enough. If *myo-fem* is truly haploinsufficient, this opens the door for the development of strong synthetic sex distorters for suppressing *Ae*. *aegypti* populations [22,55–61]. Despite the lack of female specificity for the third gene, *Aeflightin*, we reason that this still represents a useful target so long as a male-specific rescue can be performed to fully restore male flight.

Flightless *Ae*. *aegypti* have been developed previously through the transgenic overexpression of the tTa transactivator specifically in the female flight muscle [62]. In this case, the promoter region of *AeAct-4* was used to control transgene expression, however transgenic males were found to have decreased mating competitiveness in field cage trials [63,64]. Variability in the level of transgene overexpression also resulted in incomplete penetrance of the flightless phenotype. In our case, disruption of both the *AeAct-4* gene and the *myo-fem* gene through heritable gene editing resulted in a completely penetrant phenotype without the requirement for continuous transgene expression as previously seen with *AeAct-4*. As both *AeAct-4* and *myo-fem* show low levels of transcription in male pupae [51], we hypothesized that disruption of both genes would lead to reduced flight in males beyond observable differences, such as mating competitiveness. Indeed, males with mutations in *AeAct-4* exhibit decreased levels of mating when compared to wild type males. Though this decrease was not observed in males with mutations in *myo-fem*, we interpret these data with caution due to the confined nature of the mating experiment (170 oz. container), and that despite outcrossing mutants for multiple generations, there is a possibility of a genetic bottleneck leading to reduced fitness in males, regardless of these flight gene knockouts. More rigorous follow-up experiments in larger venues that require more flight effort from test males (in larger quantities and ratios of wild type to homozygous) are likely required to conclude that *myo-fem* is dispensable for male mating success. Male and female *Ae*. *aegypti* are known to produce different flight tones, which are shifted to match frequencies during mating; this harmonic convergence could be a measure of male reproductive fitness [65–68] and could be heritable [69]. It is plausible that *AeAct-4* and *myo-fem* contribute towards modulating wing beat frequency in males; future experiments to investigate this using the mutant strains developed here may shed light on this interesting aspect of *Ae*. *aegypti* biology.

**Fig 6:**
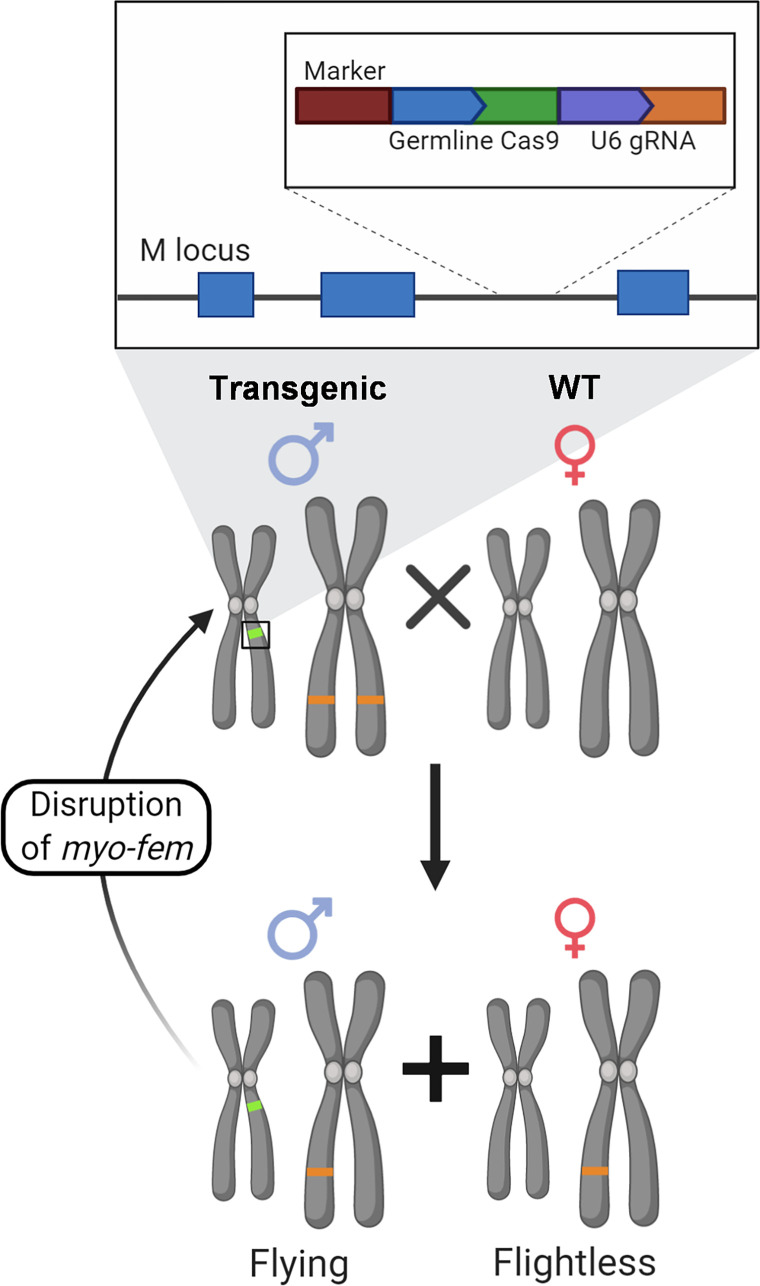
An M-locus-linked sex distorter gene drive targeting female-specific flight genes. Male mosquitoes modified to contain a site specific nuclease targeting a haploinsufficient female flight gene (*myo-fem*) in the M-locus would be released to mate with wild type females. All male progeny from these matings would inherit the nuclease, which would inactivate the intact female flight gene inherited from the mother. All female progeny from these matings would inherit one disrupted allele of the female-specific flight gene and therefore be unable to fly, blood feed, or mate to produce future offspring.

Disrupting flight specifically in female mosquitoes could be used to achieve sex ratio distortion of the adult population. This is conceptually similar to other sex distortion approaches such as the X-shredding system based on the I-PpoI homing endonuclease when active only during male meiosis, which has shown to be capable of producing >95% male progeny [56,60,70,71] and has been introgressed from *An*. *gambiae* to *An*. *arabiensis* [72]. Other examples of targets for sex ratio distortion in *An*. *gambiae* include female reproductive genes [29,57–59] and the female transcript of *doublesex*, which causes an intersex phenotype and complete sterility [59], however the recessive nature of these phenotypes reduces their power as sex distorters. In *Drosophila melanogaster*, disruption of other genes causing female fertility or embryonic lethality [22] have been shown to skew the sex ratio towards males, as does knockdown of *tra-2* in *Ae*. *aegypti* [61]. Meanwhile, overexpression of the male sex determining factor *nix* has been proposed as a method of sex distortion in *Ae*. *aegypti* [55]. Population modeling has also been evaluated when considering resistance that can evolve when using a driving Y for sex ratio distortion [73]. We note that the development of a sex ratio distortion approach that targets female-specific flight would allow for maximum competition for resources during larval development as well as allow active monitoring of the number of females doomed to flightlessness, while at the same time preventing the adult female from reproducing and potentially transmitting deadly pathogens. A gene drive-based sex ratio distortion approach targeting these female-specific flight genes could be implemented if males were carriers of the gene drive and released into the wild to mate with females, such that all female progeny would be affected and not survive to blood feed and transmit pathogens; all male progeny would inherit the transgene and continue to survive and mate at each subsequent generation (Fig 6). Such strategies to target sex-specific flight genes are likely to be applicable to other species of mosquito including Culex and Anopheles, as these mosquitoes also appear to encode sex-specific flight genes.

## Supporting information

S1 Fig Developmental expression profile of *Ae*. *aegypti* actin, myosin, and flightin genes. Heat map showing the expression of *AeAct-4*, *myo-fem*, and *Aeflightin*, as well as all paralogs with ≥80% amino acid similarity. Gene names/identifiers are listed on the right, with the developmental time points indicated above, as described by Akbari et al. [51]. Scale represents absolute expression as log_10_ (FPKM+1).
(TIF)

S2 Fig Alignment of *AeAct-4* and paralogs for sgRNA design. Nucleotide alignment of *AeAct-4* and eight paralogs with >80% nucleotide similarity. Identical nucleotides at each position are highlighted in blue; the gene model above the alignment shows the exon (box)/intron (line) boundary. Included at the top of the alignment are three sgRNAs that induced disruptions in *AeAct-4*, with the PAM sites emphasized in underlined red text.
(TIF)

S3 Fig Locations and sequencing results of closely linked paralogs to *AeAct-4*. (**A**) Three actin paralogs located near *AeAct-4* on chromosome three that were of interest to check for off-target effects. Sanger sequencing results focused around the hypothesized sgRNA target areas (indicated with a red box) based on the actin paralog alignment for AAEL001928 (**B**), AAEL005961 (**C**), and AAEL005964 (**D**).
(TIF)

S1 Table Primer sequences. Oligonucleotide sequences used for PCR amplification of each gene.
(DOCX)

S2 Table Guide RNA sequences. Oligonucleotide sequences used to synthesize sgRNAs for CRISPR-editing of each gene.
(DOCX)

S3 Table Generation of loss-of-function mutants in *Ae*. *aegypti* flight genes using CRISPR/Cas9. Raw data from embryonic injections of CRISPR/Cas9 reagents targeting each gene.
(DOCX)

S4 Table Phenotypic and genotypic analysis of *AeAct-4* and *myo-fem* G_6_ individuals. Raw data obtained following intercross of heterozygous individuals with both phenotypic (flying vs. flightless) and genotypic analysis.
(DOCX)

S5 Table Phenotypic and genotypic analysis of *Aeflightin* G_6_ individuals. Raw data obtained following intercross of heterozygous individuals with phenotypic (flying vs. flightless, and white-eyed vs. black-eyed) and genotypic analysis.
(DOCX)

S6 Table Mating competition assay between wild type and *AeAct-4*^*Δ10/Δ10*^ or *myo-fem*^*Δ11/Δ11*^ males. Raw data for each replicate, along with the mean of all replicates, for the expected and observed progeny genotypes based on male matings for each gene.
(DOCX)

S1 Video Flight tests for *AeAct-4*. Flightless *AeAct-4* individuals on the left, and control individuals on the right. The plastic buckets used to contain the adult mosquitoes were agitated by knocking on either side.
(MP4)

S2 Video Flight tests for *myo-fem*. Flightless *myo-fem* individuals on the left, and control individuals on the right. The plastic buckets used to contain the adult mosquitoes were agitated by knocking on either side.
(MP4)

S3 Video Flight tests for *Aeflightin*. Flightless *Aeflightin* individuals on the left, and control individuals on the right. The plastic buckets used to contain the adult mosquitoes were agitated by knocking on either side.
(MP4)
