# Supplementary figures and images for: CRISPR/Cas9 knockout of female-biased genes AeAct-4 or myo-fem in Ae. aegypti results in a flightless phenotype in female, but not male mosquitoes
Source: PLoS Negl Trop Dis. 2020 Dec 18;14(12):e0008971. doi: 10.1371/journal.pntd.0008971 (PMC7781531; doi:10.1371/journal.pntd.0008971)

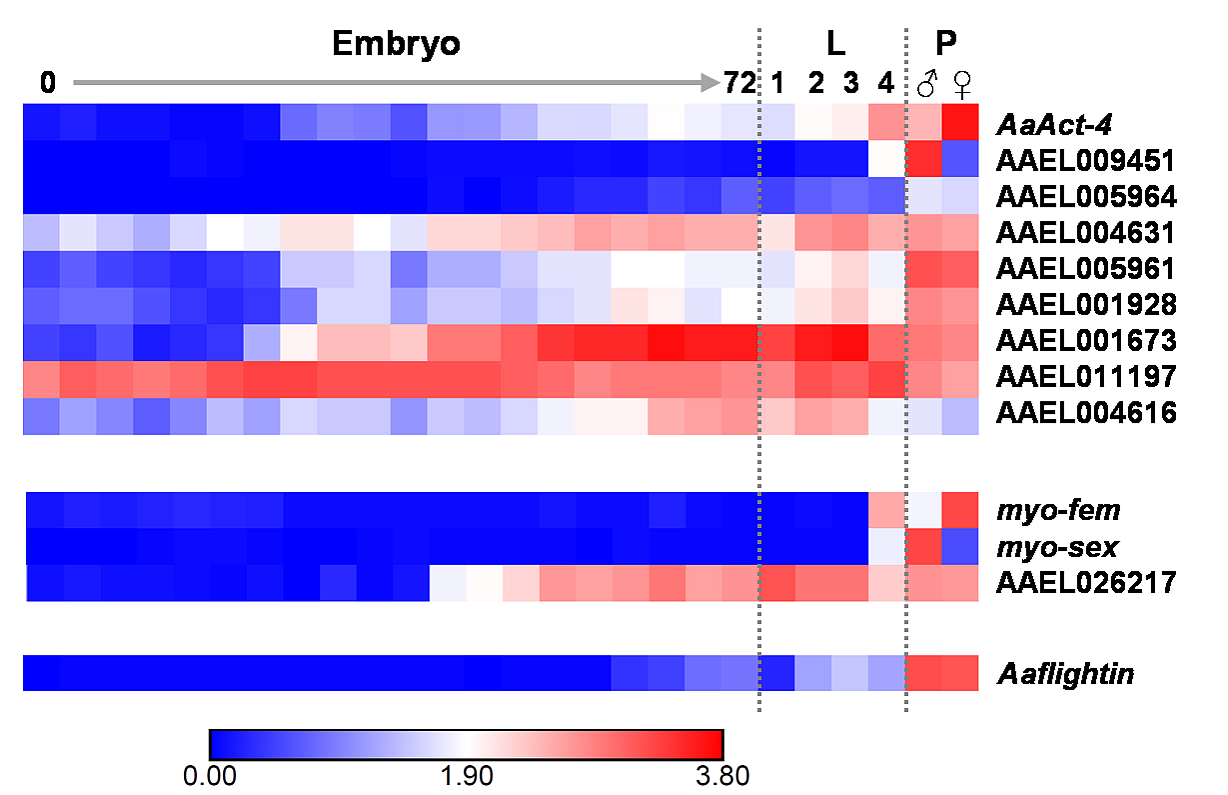

Supplement: S1 Fig — Heat map showing the expression of AeAct-4, myo-fem, and Aeflightin, as well as all paralogs with ≥80% amino acid similarity. Gene names/identifiers are listed on the right, with the developmental time points indicated above, as described by Akbari et al. [51]. Scale represents absolute expression as log10 (FPKM+1). (TIF) [file pntd.0008971.s001.tif]

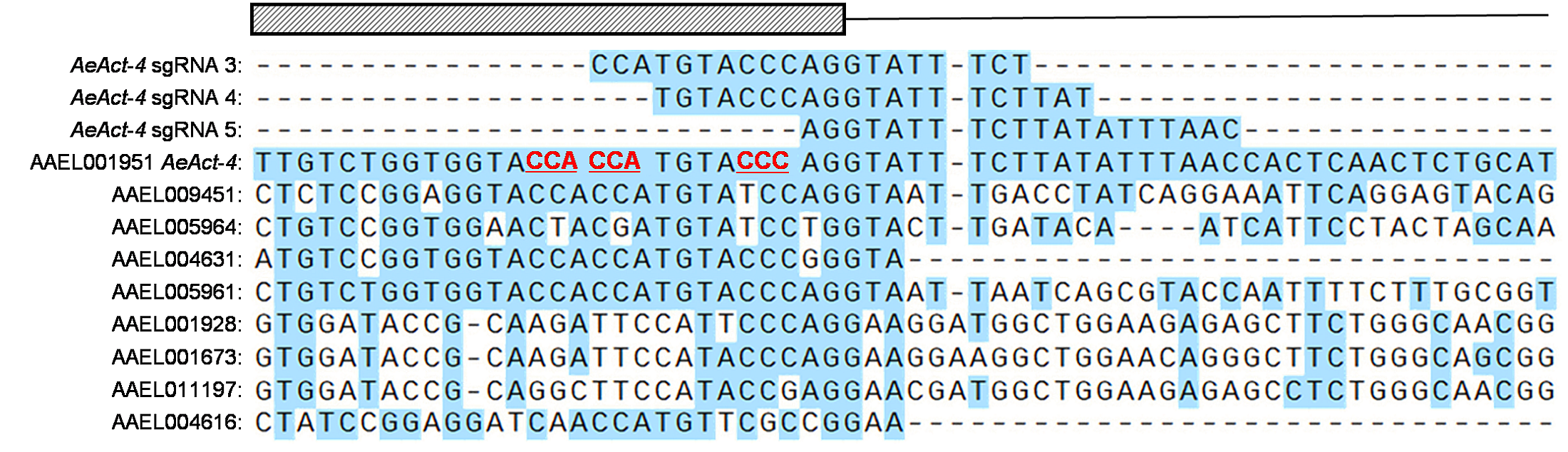

Supplement: S2 Fig — Nucleotide alignment of AeAct-4 and eight paralogs with >80% nucleotide similarity. Identical nucleotides at each position are highlighted in blue; the gene model above the alignment shows the exon (box)/intron (line) boundary. Included at the top of the alignment are three sgRNAs that induced disruptions in AeAct-4, with the PAM sites emphasized in underlined red text. (TIF) [file pntd.0008971.s002.tif]

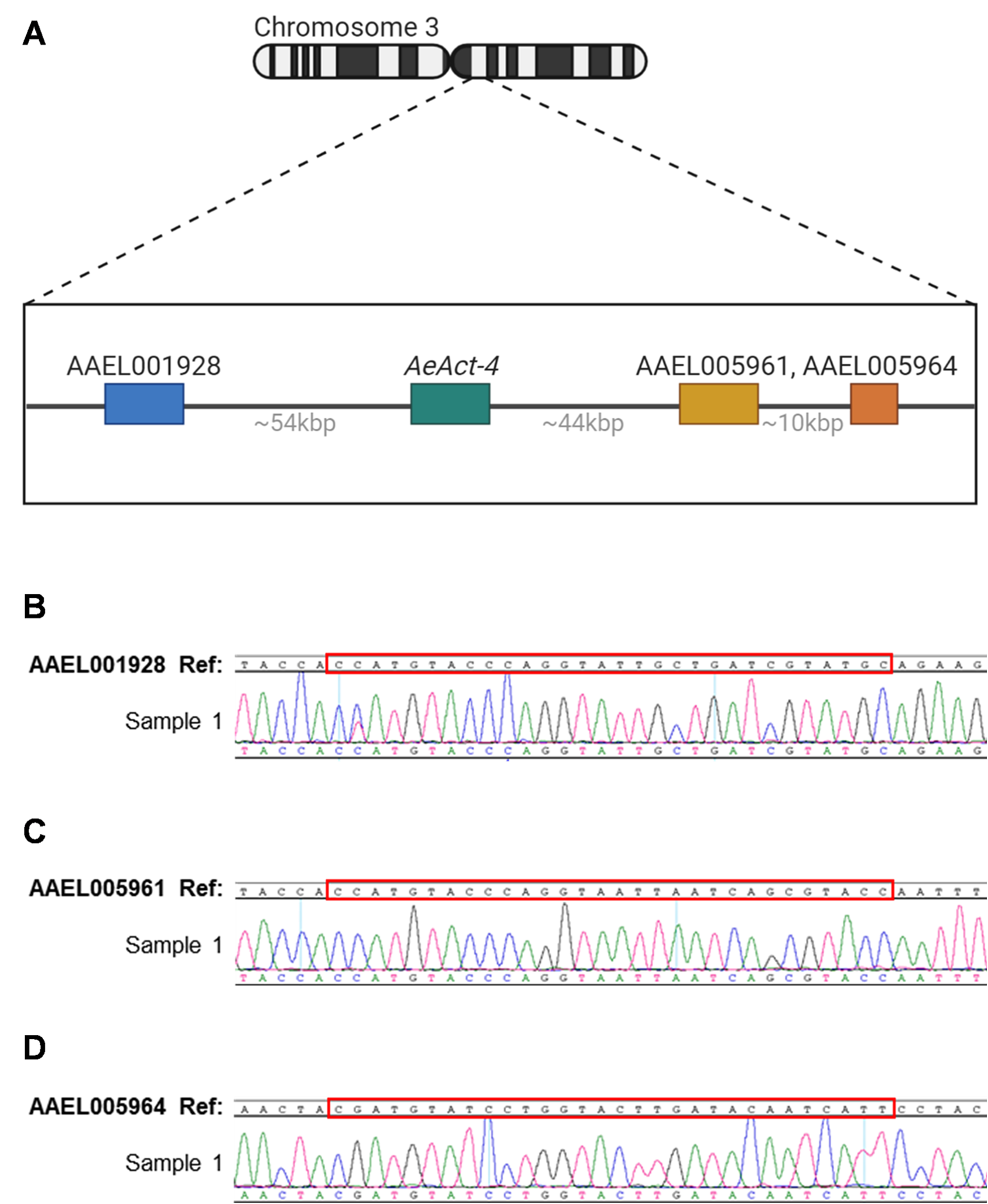

Supplement: S3 Fig — (A) Three actin paralogs located near AeAct-4 on chromosome three that were of interest to check for off-target effects. Sanger sequencing results focused around the hypothesized sgRNA target areas (indicated with a red box) based on the actin paralog alignment for AAEL001928 (B), AAEL005961 (C), and AAEL005964 (D). (TIF) [file pntd.0008971.s003.tif]
