# Supplementary material for: CRISPR/Cas9 knockout of female-biased genes AeAct-4 or myo-fem in Ae. aegypti results in a flightless phenotype in female, but not male mosquitoes
Source: PLoS Negl Trop Dis. 2020 Dec 18;14(12):e0008971. doi: 10.1371/journal.pntd.0008971 (PMC7781531; doi:10.1371/journal.pntd.0008971)
Supplement: S1 Table — Oligonucleotide sequences used for PCR amplification of each gene. (DOCX) [file pntd.0008971.s004.docx]

**S1 Table. Primer sequences.** Oligonucleotide sequences used for PCR amplification of each gene

| **Gene** | **Sequence** | **Primer use/location** |
| --- | --- | --- |
| *AeAct-4* | CCACCGCTGAACGTGAAATCGTTCG | Primer, embryo assay, site A |
| *AeAct-4* | AGAAATACCTGGGTACATGGTG | Primer, embryo assay, site A |
| *AeAct-4* | GGATCTCTATGCTAACAGCGTCTTGTC | Primer, embryo assay, site B |
| *AeAct-4* | TAGCTTGGAAGGTAGACAGC | Primer, embryo assay, site B |
| *AeAct-4* | ATCCTTCCTGGGAATGGAATCAACT | Primer, site B |
| *AeAct-4* | CTGCTTGGAGATCCACATAGCT | Primer, site B |
| *myo-fem* | TATACTTACATAGATCAGCC | Primer, exon 3 |
| *myo-fem* | TGCCGCAGACCAAGGATTTC | Primer, exon 3 |
| *myo-fem* | CCTGATCTTCCAGGGACGGC | Primer, exon 4 |
| *myo-fem* | TGTACACGTTGCGAGTCGCC | Primer, exon 4 |
| *myo-fem* | CACCGACGGCATTGTAGACC | Primer, exon 7 |
| *myo-fem* | GAAGCCTTCGATATCTTAGG | Primer, exon 7 |
| *Aeflightin* | GTTCGACTACCAACTCACCG | Primer, exon 2 |
| *Aeflightin* | TTCAGAAATAAGCGCTCGTG | Primer, exon 2 |
| *Aeflightin* | CTCTCAGTTCGCAGGACACG | Primer, exon 3 |
| *Aeflightin* | ATGAAACAAATTACAGCCCG | Primer, exon 3 |
| *Aeflightin* | GCTGTTAAGATAGCGCTTCG | Primer, exon 4 |
| *Aeflightin* | ACTAATTCAAGTGAACTCAC | Primer, exon 4 |
| AAEL001928 | ATCCTTCCTGGGAATGGAATCAACT | Primer |
| AAEL001928 | CCTATTCTTCAGGATTAACTTAGAAGC | Primer |
| AAEL005961 | ATCCTTCCTGGGAATGGAATCTGCT | Primer |
| AAEL005961 | CGCAAAGAAAATTGGTACGC | Primer |
| AAEL005964 | ATCGTTCTTGGGCATGGAAACGGCA | Primer |
| AAEL005964 | GGTGCAACGGCGGTTATCTCT | Primer |
