# Supplementary material for: CRISPR/Cas9 knockout of female-biased genes AeAct-4 or myo-fem in Ae. aegypti results in a flightless phenotype in female, but not male mosquitoes
Source: PLoS Negl Trop Dis. 2020 Dec 18;14(12):e0008971. doi: 10.1371/journal.pntd.0008971 (PMC7781531; doi:10.1371/journal.pntd.0008971)
Supplement: S2 Table — Oligonucleotide sequences used to synthesize sgRNAs for CRISPR-editing of each gene. (DOCX) [file pntd.0008971.s005.docx]

**S2 Table. Guide RNA sequences.** Oligonucleotide sequences used to synthesize sgRNAs for CRISPR-editing of each gene.

| **Gene** | **Sequence** | **Guide RNA location** |
| --- | --- | --- |
| *AeAct-4* | GAAATTAATACGACTCACTATAGG **GGGAAGTTCATAAGACTTCT** GTTTTAGAGCTAGAAA | Guide RNA, site A |
| *AeAct-4* | GAAATTAATACGACTCACTATAGG **ATCAACTGGCATTCATGAAA** GTTTTAGAGCTAGAAA | Guide RNA, site A |
| *AeAct-4* | GAAATTAATACGACTCACTATAGG **AGAAATACCTGGGTACATGG** GTTTTAGAGCTAGAAA | Guide RNA, site B |
| *AeAct-4* | GAAATTAATACGACTCACTATAGG **ATAAGAAATACCTGGGTACA** GTTTTAGAGCTAGAAA | Guide RNA, site B |
| *AeAct-4* | GAAATTAATACGACTCACTATAGG **GTTAAATATAAGAAATACCT** GTTTTAGAGCTAGAAA | Guide RNA, site B |
| *myo-fem* | GAAATTAATACGACTCACTATAGG **TCTCAAGTTATGTAACACAG** GTTTTAGAGCTAGAAA | Guide RNA, exon 3 |
| *myo-fem* | GAAATTAATACGACTCACTATAGG **GTAACACAGAGGCATCGTTG** GTTTTAGAGCTAGAAA | Guide RNA, exon 3 |
| *myo-fem* | GAAATTAATACGACTCACTATAGG **CACAGAGGCATCGTTGAGGT** GTTTTAGAGCTAGAAA | Guide RNA, exon 3 |
| *myo-fem* | GAAATTAATACGACTCACTATAGG **AAAAACTGAAAACACAAAGA** GTTTTAGAGCTAGAAA | Guide RNA, exon 4 |
| *myo-fem* | GAAATTAATACGACTCACTATAGG **TTTTCCTGCACCAGACTCGC** GTTTTAGAGCTAGAAA | Guide RNA, exon 4 |
| *myo-fem* | GAAATTAATACGACTCACTATAGG **ATTACCGGCGAGTCTGGTGC** GTTTTAGAGCTAGAAA | Guide RNA, exon 4 |
| *myo-fem* | GAAATTAATACGACTCACTATAGG **ATGTTGATTACCGGCGAGTC** GTTTTAGAGCTAGAAA | Guide RNA, exon 4 |
| *myo-fem* | GAAATTAATACGACTCACTATAGG **TAAACTGCTGGGTTGCGTTA** GTTTTAGAGCTAGAAA | Guide RNA, exon 7 |
| *myo-fem* | GAAATTAATACGACTCACTATAGG **AACCCAGCAGTTTAGCGACT** GTTTTAGAGCTAGAAA | Guide RNA, exon 7 |
| *myo-fem* | GAAATTAATACGACTCACTATAGG **GACCGAGTCGCTAAACTGCT** GTTTTAGAGCTAGAAA | Guide RNA, exon 7 |
| *myo-fem* | GAAATTAATACGACTCACTATAGG **GTCACCTTCTTCTATGCCAT** GTTTTAGAGCTAGAAA | Guide RNA, exon 7 |
| *Aeflightin* | GAAATTAATACGACTCACTATAGG **AAGTCATCGACAATGTCTAG** GTTTTAGAGCTAGAAA | Guide RNA, exon 2 |
| *Aeflightin* | GAAATTAATACGACTCACTATAGG **TGTCTAGCGGCTCCGCTCCG** GTTTTAGAGCTAGAAA | Guide RNA, exon 2 |
| *Aeflightin* | GAAATTAATACGACTCACTATAGG **GGTGAAAAATACCCGCGGAG** GTTTTAGAGCTAGAAA | Guide RNA, exon 2 |
| *Aeflightin* | GAAATTAATACGACTCACTATAGG **AGACGGGCTCGCGGAATGCC** GTTTTAGAGCTAGAAA | Guide RNA, exon 3 |
| *Aeflightin* | GAAATTAATACGACTCACTATAGG **TTCCGCGAGCCCGTCTGTCC** GTTTTAGAGCTAGAAA | Guide RNA, exon 3 |
| *Aeflightin* | GAAATTAATACGACTCACTATAGG **TGATGACGTCATAGATTACC** GTTTTAGAGCTAGAAA | Guide RNA, exon 3 |
| *Aeflightin* | GAAATTAATACGACTCACTATAGG **ATCCCGCTTCAATCCGATGT** GTTTTAGAGCTAGAAA | Guide RNA, exon 4 |
| *Aeflightin* | GAAATTAATACGACTCACTATAGG **ATGTTGGTGTAGTTGTACAT** GTTTTAGAGCTAGAAA | Guide RNA, exon 4 |
| *Aeflightin* | GAAATTAATACGACTCACTATAGG **TACATTTCGTCGATGCTCTT** GTTTTAGAGCTAGAAA | Guide RNA, exon 4 |
