# Supplementary material for: CRISPR/Cas9 knockout of female-biased genes AeAct-4 or myo-fem in Ae. aegypti results in a flightless phenotype in female, but not male mosquitoes
Source: PLoS Negl Trop Dis. 2020 Dec 18;14(12):e0008971. doi: 10.1371/journal.pntd.0008971 (PMC7781531; doi:10.1371/journal.pntd.0008971)
Supplement: S3 Table — Raw data from embryonic injections of CRISPR/Cas9 reagents targeting each gene. (DOCX) [file pntd.0008971.s006.docx]

**S3 Table. Generation of loss-of-function mutants in *Ae. aegypti* flight genes using CRISPR/Cas9.** Raw data from embryonic injections of CRISPR/Cas9 reagents targeting from each gene.

| **Line** | **# Injected** | **# G_0_ Hatched** | **% G_0_ Survival** | **# G_1_ Genotyped** | **# G_1_ Sequenced** | **# G_1_ Mutants** | **Results** |
| --- | --- | --- | --- | --- | --- | --- | --- |
| *AeAct-4* Exp. 1 | 407 | 83 | 20.4% | 240 | 19 | 2 | Δ3 |
| *myo-fem* Exon 3 Exp. 1 | 468 | 43 | 9.2% | 320 | 20 | 9 | Δ4Δ4, Δ3, i2, Δ11 |
| *myo-fem* Exon 4 Exp. 1 | 455 | 54 | 11.9% | 197 | 6 | 0 | N/A |
| *Aeflightin* Exon 2 Exp. 1 | 373 | 49 | 13.1% | 80 | 10 | 2 | Δ9 |
| *Aeflightin* Exon 4 Exp. 1 | 353 | 57 | 16.2% | 240 | 4 | 2 | Δ3 |
| *myo-fem* Exon 4 Exp. 2 | 265 | 29 | 11.0% | 80 | 0 | 0 | N/A |
| *Aeflightin* Exon 2 Exp. 2 | 362 | 114 | 31.5% | 240 | 23 | 15 | Δ2Δ5a, Δ5b, Δ4, Δ2Δ5b, Δ5c |
| *Aeflightin* Exon 4 Exp. 2 | 268 | 58 | 21.6% | 80 | 0 | 0 | N/A |
| *AeAct-4* B Exp. 2 | 268 | 66 | 24.6% | 80 | 10 | 4 | Δ2, Δ10 |
