# Supplementary material for: CRISPR/Cas9 knockout of female-biased genes AeAct-4 or myo-fem in Ae. aegypti results in a flightless phenotype in female, but not male mosquitoes
Source: PLoS Negl Trop Dis. 2020 Dec 18;14(12):e0008971. doi: 10.1371/journal.pntd.0008971 (PMC7781531; doi:10.1371/journal.pntd.0008971)
Supplement: S4 Table — Raw data obtained following intercross of heterozygous individuals with both phenotypic (flying vs. flightless) and genotypic analysis. (DOCX) [file pntd.0008971.s007.docx]

**S4 Table. Phenotypic and genotypic analysis of *AeAct-4* and *myo-fem* G_6_ individuals.** Raw data obtained following intercross of heterozygous individuals with both phenotypic (flying vs. flightless) and genotypic analysis.

| ***AeAct-4* G_5_:** | | **♂ Δ10/+ X ♀ Δ10/+** | | | |  | |  | |
| --- | --- | --- | --- | --- | --- | --- | --- | --- | --- |
| **Flying:**  222/251 (88.4%) | | | | **Flightless:**  29/251 (11.6%) | | | | | |
|  | **Male:** | **Female:** | |  | | **Male:** | | **Female:** | |
| **Δ10/Δ10:** | 35/124 (28.2%) | 0 | | **Δ10/Δ10:** | | 0 | | 27/29 (93.1%) | |
| **Δ10/+:** | 61/124 (49.2%) | 69/98 (70.4%) | | **Δ10/+:** | | 0 | | 2/29 (6.9%) | |
| **+/+:** | 28/124 (22.6%) | 29/98 (29.6%) | | **+/+:** | | 0 | | 0 | |
| ***myo-fem* G_5_:** | | | **♂ Δ11/+ X ♀ Δ11/+** | | | |  | |  |
| **Flying:**  238/314 (75.8%) | | | | | **Flightless:**  76/314 (24.2%) | | | | |
|  | **Male:** | | **Female:** | |  | | **Male:** | | **Female:** |
| **Δ11/Δ11:** | 24/158 (15.2%) | | 0 | | **Δ11/Δ11:** | | 1/2 (50.0%) | | 28/74 (37.8%) |
| **Δ11/+:** | 102/158 (64.6%) | | 41/80 (51.3%) | | **Δ11/+:** | | 1/2 (50.0%) | | 46/74 (62.2%) |
| **+/+:** | 32/158 (20.3%) | | 39/80 (48.8%) | | **+/+:** | | 0 | | 0 |
