# Supplementary material for: CRISPR/Cas9 knockout of female-biased genes AeAct-4 or myo-fem in Ae. aegypti results in a flightless phenotype in female, but not male mosquitoes
Source: PLoS Negl Trop Dis. 2020 Dec 18;14(12):e0008971. doi: 10.1371/journal.pntd.0008971 (PMC7781531; doi:10.1371/journal.pntd.0008971)
Supplement: S5 Table — Raw data obtained following intercross of heterozygous individuals with phenotypic (flying vs. flightless, and white-eyed vs. black-eyed) and genotypic analysis. (DOCX) [file pntd.0008971.s008.docx]

**S5 Table. Phenotypic and genotypic analysis of *Aeflightin* G_6_ individuals.** Raw data obtained following intercross of heterozygous individuals with phenotypic (flying vs. flightless, and white-eyed vs. black-eyed) and genotypic analysis.

| ***Aeflightin* G_5_:** | | | ♂ *Aeflightin*^Δ4/+^*kmo*^+/-^ X ♀ *Aeflightin*^Δ5/+^*kmo*^+/-^ | | | | | |
| --- | --- | --- | --- | --- | --- | --- | --- | --- |
| **Flying, white-eyed:**  80/80 (100%) | | | **Flying, black-eyed:**  315/463 (68.0%) | | | **Flightless, black-eyed:**  148/463 (32.0%) | | |
|  | **Male:** | **Female:** |  | **Male:** | **Female:** |  | **Male:** | **Female:** |
| **Δ4/Δ5:** | 0 | 0 | **Δ4/Δ5:** | 0 | 0 | **Δ4/Δ5:** | 48/52 (92.3%) | 91/96 (94.8%) |
| **Δ5/+:** | 0 | 1/40 (2.5%) | **Δ5/+:** | 79/156 (50.6%) | 69/159 (43.4%) | **Δ5/+:** | 0 | 3/96 (3.1%) |
| **Δ4/+:** | 1/40 (2.5%) | 0 | **Δ4/+:** | 74/156 (47.4%) | 90/159 (56.6%) | **Δ4/+:** | 4/52 (7.7%) | 2/96 (2.1%) |
| **+/+:** | 39/40 (97.5%) | 39/40 (97.5%) | **+/+:** | 3/156 (1.9%) | 0 | **+/+:** | 0 | 0 |
