# Supplementary material for: CRISPR/Cas9 knockout of female-biased genes AeAct-4 or myo-fem in Ae. aegypti results in a flightless phenotype in female, but not male mosquitoes
Source: PLoS Negl Trop Dis. 2020 Dec 18;14(12):e0008971. doi: 10.1371/journal.pntd.0008971 (PMC7781531; doi:10.1371/journal.pntd.0008971)
Supplement: S6 Table — Raw data for each replicate, along with the mean of all replicates, for the expected and observed progeny genotypes based on male matings for each gene. (DOCX) [file pntd.0008971.s009.docx]

**S6 Table. Mating competition assays between wild type and *AeAct-4^Δ10/Δ10^* or *myo-fem^Δ11/Δ11^* males.** Raw data for each replicate along with the mean of all replicates for the expected and observed progeny genotypes based on male matings for each gene.

| **Gene** | **# WT Males (%)** | **# HET Males (%)** | **# HOM Males (%)** | **# Total Males** | ***Total Matings** | **# Exp. WT (%)** | **# Exp. HET (%)** | **# Obs. WT (%)** | **# Obs. HET (%)** | **χ2** | **P-value** |
| --- | --- | --- | --- | --- | --- | --- | --- | --- | --- | --- | --- |
| *AeAct-4* Rep 1 | 16 (40%) | 1 (3%) | 23 (58%) | 40 | 26 | 10 (38%) | 16 (62%) | 17 (65%) | 9 (35%) | 7.963 | 0.0048 |
| *AeAct-4* Rep 2 | 23 (58%) | 0 (0%) | 17 (43%) | 40 | 35 | 20 (57%) | 15 (43%) | 30 (86%) | 5 (14%) | 11.667 | 0.0006 |
| *AeAct-4* Rep 3 | 16 (40%) | 1 (3%) | 23 (58%) | 40 | 32 | 13 (41%) | 19 (59%) | 22 (69%) | 10 (31%) | 10.494 | 0.0012 |
| *AeAct-4* Mean |  | | | | | 14 | 17 | 23 | 8 |  | |
| *myo-fem* Rep 1 | 21 (53%) | 4 (10%) | 15 (38%) | 40 | 27 | 14 (52%) | 13 (48%) | 21 (78%) | 6 (22%) | 7.269 | 0.0070 |
| *myo-fem* Rep 2 | 15 (38%) | 0 (0%) | 25 (63%) | 40 | 11 | 4 (36%) | 7 (64%) | 10 (91%) | 1 (9%) | 14.143 | 0.0002 |
| *myo-fem* Rep 3 | 20 (50%) | 0 (0%) | 20 (50%) | 40 | 31 | 16 (50%) | 16 (50%) | 15 (48%) | 16 (52%) | 0.032 | 0.8575 |
| *myo-fem* Rep 4 | 20 (50%) | 0 (0%) | 20 (50%) | 40 | 16 | 8 (50%) | 8 (50%) | 10 (63%) | 6 (38%) | 1.000 | 0.3173 |
| *myo-fem* Mean |  | | | | | 10 | 11 | 14 | 7 |  | |

*Matings are defined as females who blood fed, laid embryos, and hatched.
